# Spread and Scale of the Integrated Nutrition Pathway for Acute Care Across Canada: Protocol for the Advancing Malnutrition Care Program

**DOI:** 10.2196/62764

**Published:** 2024-12-31

**Authors:** Katherine L Ford, Celia Laur, Rupinder Dhaliwal, Roseann Nasser, Leah Gramlich, Johane P Allard, Heather Keller, AMC Core Team

**Affiliations:** 1 Department of Kinesiology and Health Sciences University of Waterloo Waterloo, ON Canada; 2 Women’s College Hospital Institute for Health System Solutions and Virtual Care Toronto, ON Canada; 3 Institute of Health Policy, Management and Evaluation University of Toronto Toronto, ON Canada; 4 Canadian Malnutrition Task Force Canadian Nutrition Society Kemptville, ON Canada; 5 Clinical Nutrition Services Saskatchewan Health Authority Regina, SK Canada; 6 Department of Medicine University of Alberta Edmonton, AB Canada; 7 Department of Medicine University of Toronto Toronto, ON Canada; 8 Schlegel-University of Waterloo Research Institute for Aging Waterloo, ON Canada; 9 See Acknowledgements

**Keywords:** malnutrition, nutrition screening, nutrition assessment, hospital, malnutrition care, nutrition, acute care, clinicians, mixed-methods design, decision making, mentor-champion model, virtual training, peer support, virtual community of practice

## Abstract

**Background:**

A high proportion of patients admitted to hospital are at nutritional risk or have malnutrition. However, this risk is often not identified at admission, which may result in longer hospital stays and increased likelihood of death. The Integrated Nutrition Pathway for Acute Care (INPAC) was developed to provide clinicians with a standardized approach to prevent, detect, and treat malnutrition in hospital.

**Objective:**

The purpose of this study was to determine if the Advancing Malnutrition Care (AMC) program can be used to spread and scale-up improvements to nutrition care in Canadian hospitals.

**Methods:**

A prospective, longitudinal, mixed methods design is proposed to evaluate the spread and scale of INPAC best practices across Canadian hospitals using a mentor-champion model. Purposive and snowball sampling are used to recruit mentors and hospital champions to participate in the AMC program. Mentors are persons with experience improving nutrition care in a clinical setting and champions are health care providers with a commitment to implementing best care practices. Mentors and champions are trained digitally on their roles and activities. Mentors meet with champions in their area monthly to support them with making practice change. Champions created a site implementation team to target practice change in a specific area related to malnutrition care and use AMC program-specific tools and resources to implement improvements and collect site information through quarterly audits of patient charts to track implementation of nutrition care best practices. An online community of practice is held every 3-4 months to provide further implementation resources and foster connection between mentors and champions at a national level. A prospective evaluation will be conducted to assess the impact of the program and explore how it can be sustainably spread and scaled across Canada. Semistructured interviews will be used to gain a deeper understanding of mentor and champion experiences in the program. The capabilities, opportunities, and motivations of behavior model will be used to evaluate behavior change and the Kirkpatrick 4-level framework will facilitate assessment of barriers to change. Aggregated chart audits will assess the impact of implemented care practices. Descriptive analyses will be used to describe baseline mentor and champion and hospital characteristics and mentor and champion experiences; Friedman test will describe these changes over time. Directed content analysis will guide interpretation of interview data.

**Results:**

Data collection began in September 2022 and is anticipated to end in June 2025, at which time data analysis will begin.

**Conclusions:**

Evaluation of the AMC program will strengthen decision-making, future programming, and will inform program changes that reflect implementation of best practices in nutrition care while supporting regional mentors and hospital champions. This work will address the sustainability of AMC and the critical challenges related to hospital-based malnutrition, ultimately improving nutrition care for patients across Canada.

**International Registered Report Identifier (IRRID):**

DERR1-10.2196/62764

## Introduction

Disease-related malnutrition is prevalent and observed in up to 1 in 2 patients admitted to hospital in Canada [1]. Patients with malnutrition at time of admission are at increased risk of longer length of stay, increased cost of care, disability, morbidity, and mortality [1-3]. Nutrition interventions are associated with improved clinical outcomes including survival [4-6] in patients at nutrition risk or with malnutrition, yet not all patients in need receive nutrition support [1]. The problem of disease-related malnutrition is not unique to Canada; it is an ongoing clinical issue that continues to lack recognition, detection, and treatment in hospitals globally [7,8].

To identify nutrition risk and malnutrition in the hospitalized setting, all patients, regardless of age, body shape and size, should be screened [9]. Several validated tools for nutrition screening exist [10,11]; yet, screening rates vary greatly across hospitals and units [12,13]. It is recommended that patients who screen positive for nutrition risk receive a complete nutrition assessment and appropriate nutrition intervention from a registered dietitian [14], although this practice is not widely implemented [15]. Without standardized nutrition care practices, patients at nutrition risk and those with malnutrition are not identified and may not be referred to a dietitian when needed [1,8,13].

**Figure 1:**
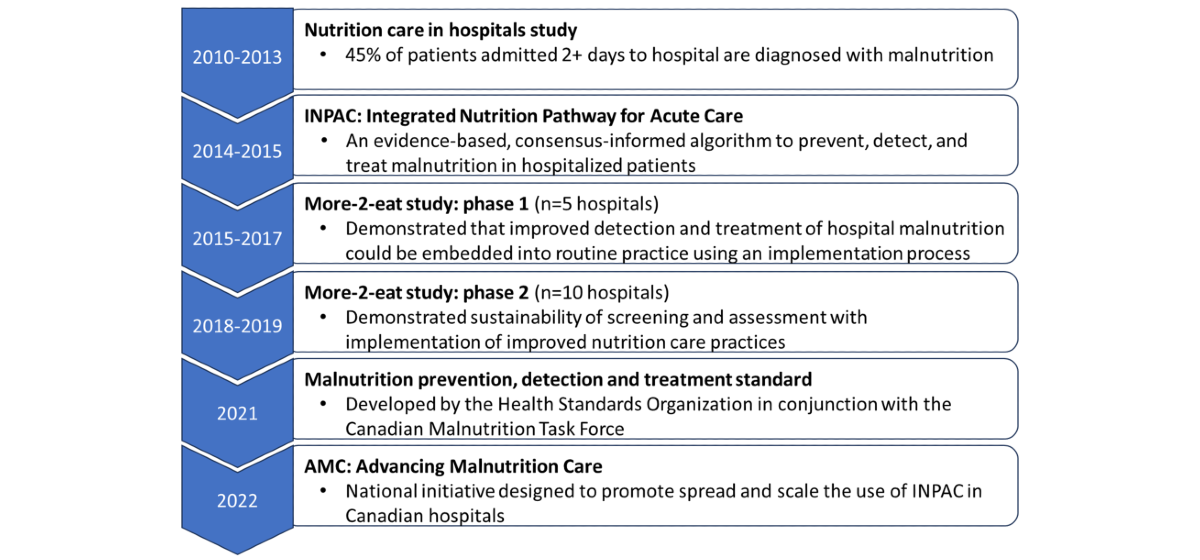
Overview of malnutrition care initiatives by the Canadian Malnutrition Task Force. INPAC: Integrated nutrition pathway for acute care.

The foundational work required to begin standardizing nutrition care practices in Canadian hospitals has been underway since 2010 (Figure 1). To improve the identification and treatment process for patients at nutrition risk or with malnutrition, best practices were developed and strategies to combat hospital malnutrition were implemented [14-20]. Specifically, the evidence-informed and consensus-based Integrated Nutrition Pathway for Acute Care (INPAC) was developed to support clinicians with addressing nutrition-related gaps in care [21]. This pathway is an algorithm based on minimum clinical practice standards required for prevention, detection, and treatment of malnutrition for patients admitted to medical and surgical units and is a means to standardize nutrition care practices related to screening, assessment, interventions and follow-up [14,22,23].

INPAC was tested for feasibility and effectiveness in 2 phases and demonstrated that medical and surgical units in hospitals across 8 Canadian provinces could implement INPAC components such as nutrition risk screening on admission (Figure 1) [15,16,24-26]. In 2021, the Health Standard Organization published a national malnutrition standard (Malnutrition Prevention, Detection, and Treatment - CAN/HSO 5066:2021(E)) [9]*.* The components of the standard are based on INPAC, recognizing the importance of systematizing nutrition care. The standard provides hospitals with best practices that support the detection, treatment, and prevention of malnutrition and improve health outcomes of hospitalized patients [9].

The Advancing Malnutrition Care (AMC) program was developed as a way to spread implementation of INPAC and scale use of the malnutrition standard to improve nutrition care practices in hospitals across Canada [27]. By spread, we are referring to the replication of INPAC implementation from one hospital to the next [28]. By scale, we are referring to development of the infrastructure to support implementation of INPAC and support national use of the malnutrition standards [28]. AMC uses a mentor-champion model to engage and train regional mentors and connect them with hospital champions to both spread and scale nutrition care best practices across Canada in a feasible and effective way. The purpose of this study is to determine if the AMC program can be used to spread and scale-up improvements to nutrition care in Canadian hospitals. The following research questions will be used to evaluate AMC:

1. Is virtual training effective to support mentor and hospital champion capability to implement improved nutrition care practices in Canadian hospitals?
2. What implementation strategies do hospital sites use and what is the impact on nutrition care practices?
3. Does the AMC program increase mentor and champion opportunity, motivation, or confidence for implementing practice change? What are mentor and champion experiences with AMC?
4. What barriers and facilitators do mentors and champions experience related to their implementation roles?
5. Does a virtual community of practice support spread and scale of AMC?
6. How does the qualitative interview data on mentor and champion experiences further explain the impact of the AMC program on the quantitively measured improved nutrition care practices?

## Methods

### Advancing Malnutrition Care Program Implementation

#### Design and Objectives

**Figure 2:**
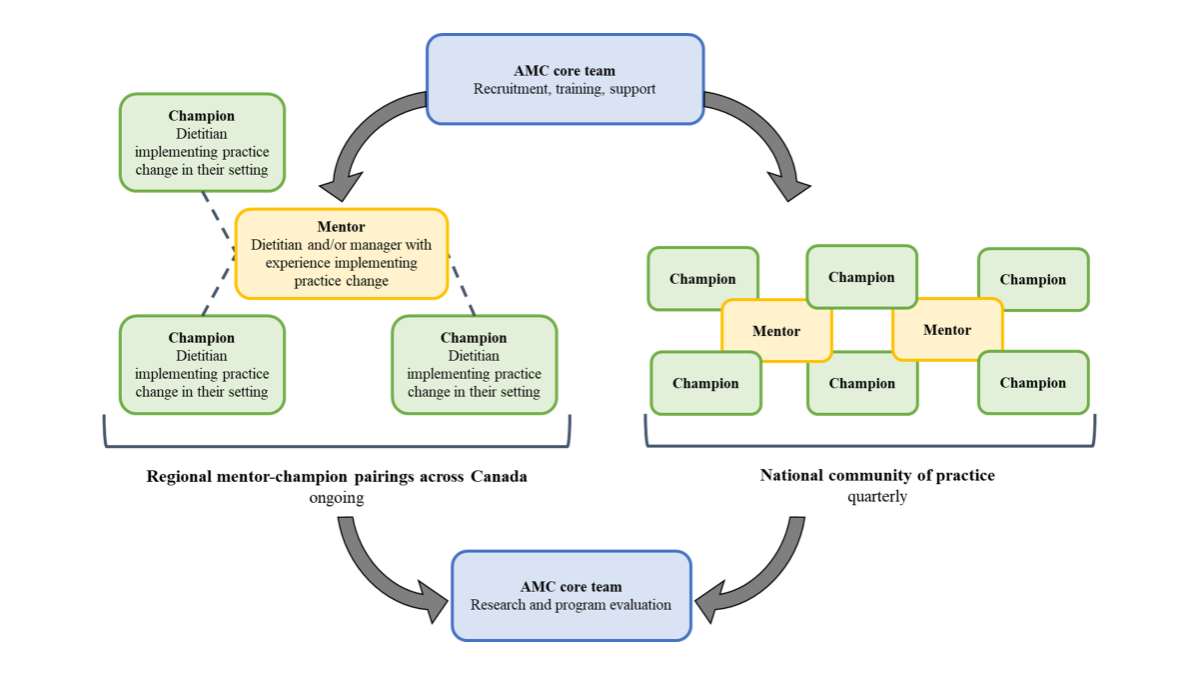
Advancing Malnutrition Care program design. AMC: Advancing Malnutrition Care.

**Figure 3:**
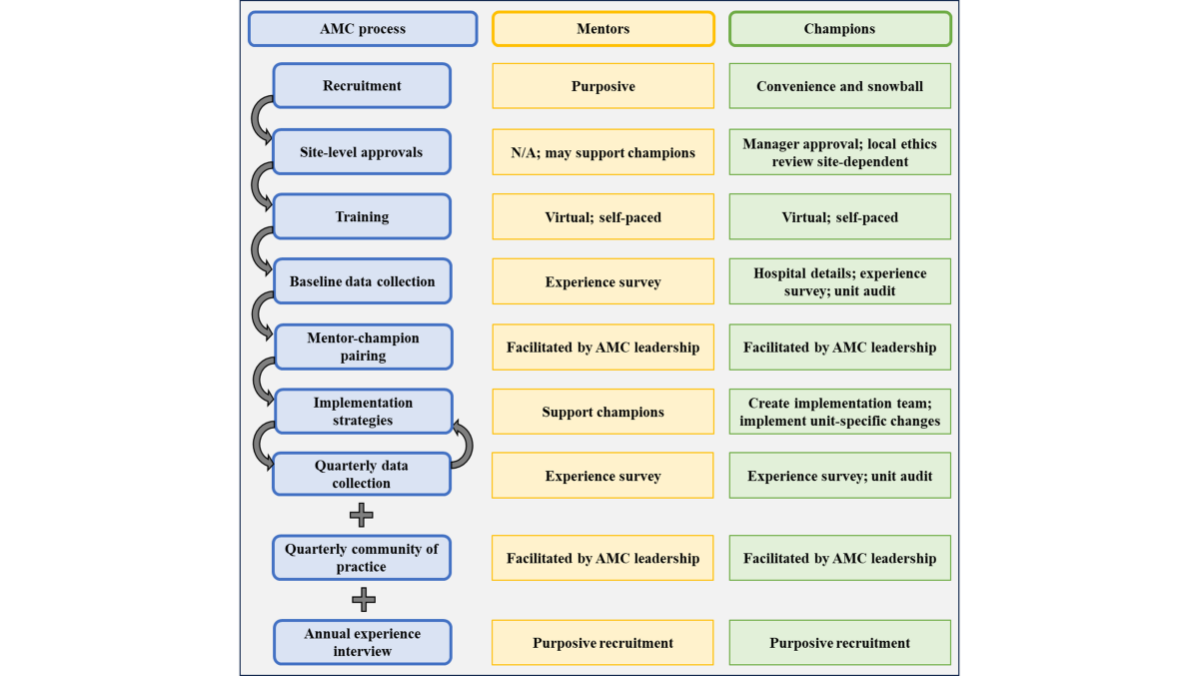
Mentor and champion flow through the Advancing Malnutrition Care program. AMC: Advancing Malnutrition Care; N/A: not applicable.

The AMC Core Team recruits, trains, and supports regional mentors, typically dietitians or managers with experience improving nutrition care in a clinical setting and connects them with hospital champions who are typically front-line clinical dietitians. The AMC Core Team supports a quarterly national community of practice to enhance mentor-champion capacity building for implementation and oversees AMC program evaluation (Figure 2). About 20 mentors and 75 hospital champions will be recruited from approximately 20 hospitals across Canada. Having 75 champions across Canadian provinces will support our understanding of a mentor-champion model among health systems in Canada. As this is an existing program being evaluated, a sample size calculation is not necessary to support the sample required to address the research questions. The flow of mentors and champions through the AMC program is described in Figure 3.

The objectives of the AMC program are to (1) virtually train regional mentors and develop their mentorship and coaching capabilities to support champions with implementing improved nutrition care practices; (2) virtually train hospital champions and develop their capacity to implement change in their practice setting; and (3) facilitate and support ongoing INPAC implementation efforts.

#### Theoretical Foundation and Frameworks Guiding the Advancing Malnutrition Care Program

An integrated knowledge translation model was used to design AMC [29]. This model uses a collaborative approach by researchers and decision makers. The knowledge to action process [30] is used to implement strategies and move through stages of the action cycle. This model was previously used with the More-2-Eat studies and is applied with AMC [15,31]. Theories of behavior change are also applied to the implementation strategies, with the overall program informed by the Behavior Change Wheel, a method for characterizing behavior change centered around sources of behavior delimited by intervention functions and policy categories [32]. The sources of behavior of this model include capabilities, opportunities, and motivations of behavior (COM-B model) and are interacting to produce behaviors that influence intervention and policy [32]. Strategies to support hospital champions with implementing INPAC in their hospital settings are drawn from the Expert Recommendations for Implementing Change taxonomy—a consensus-based compilation of implementation strategies [33]*.*

#### Recruitment and Participants

Mentors are purposefully recruited based on their previous involvement with the More-2-Eat studies or other hospital implementation experience. A recruitment email message was sent to Canadian Malnutrition Task Force networks and further disseminated to professional networks (ie, Canadian Nutrition Society) to recruit hospital champions. AMC program information and request for hospital champions is advertised on the Canadian Malnutrition Task Force website [34] and social media feeds; is presented at national conferences and webinars; and shared through snowball sampling. Enrolment is ongoing and responsive to new opportunities. Mentors may also recruit hospital champions from their networks using the general email recruitment message and poster provided by the AMC research team. Ideally, mentors will have a passion for addressing malnutrition care in a hospital setting, demonstrated commitment to successful implementation of INPAC, experience making practice changes and a keen desire to help others, enthusiasm, and capacity to support INPAC implementation, and an understanding of the local hospital and regional contexts. The role of the mentor includes receiving mentorship training from AMC leadership; supporting hospital champions in their region with implementation of INPAC; facilitating mentor-champion meetings and where necessary, one-on-one meetings; participating in quarterly national community of practice meetings; debriefing with AMC leadership at occasional AMC mentor meetings; and completing baseline and quarterly surveys. Mentors may choose to assume a dual role as mentor and hospital champion.

Ideally, hospital champions will have a passion for addressing malnutrition care; enthusiasm and capacity to drive the implementation process; an understanding of the local hospital context; and a commitment to implement best practices and participate in a mentorship model to improve hospital nutrition care. Their role includes completing training on INPAC implementation and sustainability; implementing INPAC practices in their units; receiving guidance from an AMC mentor on how best to implement INPAC; undertaking quarterly patient chart audits to assess changes in practice, and feeding back results to relevant hospital teams (eg, site implementation team); sharing progress with AMC mentors; participating in quarterly national community of practice sessions; completing baseline and quarterly surveys.

#### Training

The AMC leadership developed virtual orientation and training modules based on implementation science, behavior change theory, and real-world experience from the More-2-Eat projects [15-17]. Modules were reviewed by the AMC Core team and are used by mentors and hospital champions before the start of implementation efforts. Mentor training is focused on mentorship and behavior change, including the role of mentors in the AMC program, how to support hospital champions with implementation of INPAC, and sharing strategies for a meaningful and successful mentor-champion partnership. Mentor training is self-paced and takes approximately 2 hours to complete. Mentors have the option to review the hospital champion training materials.

Hospital champion training is focused on INPAC as an algorithm to support malnutrition care, strategies to implement INPAC, how to support behavior change, including the role of hospital champions and mentors, strategies for getting started, developing buy-in and engagement, adoption of changes into routine practice, using behavior change strategies, and collecting data and feeding it back to relevant teams, to support adoption of change in routine practice. Hospital champion training is self-paced, spread over 3 modules, and takes approximately 3 hours to complete.

Both mentors and champions complete a behavior change training module focused on how to implement practice change that aligns with INPAC. This module reviews the basics of change, with a focus on the COM-B model [32], the Behavior Change Wheel [32], and action steps related to the “Plan Do Check Act” [35] model for change.

#### Mentor-Champion Pairing

The AMC leadership pairs hospital champions with a regional (eg, health systems) mentor. The purpose of the mentor-champion pairing is for mentors to guide hospital champions through implementation efforts, advise on implementation challenges, celebrate implementation successes, and support practice change in the study unit. Based on the number of champions that are paired with a mentor, the mentor will choose to host individual or group mentor-champion meetings. These meetings occur in-person or virtually on a monthly or bimonthly basis, depending on the location of the mentor and hospital champions and their preferences and implementation needs. The AMC leadership can facilitate the first mentor-champion meeting; subsequent meetings are arranged by the mentor-champion pair.

#### Implementation

Champions are encouraged to first create a site implementation team with select individuals (eg, nurses, physicians, dietitians, food service personnel, and key management personnel) involved in the targeted practice change (eg, improving rates of nutrition risk screening at admission). The number of members and composition of this team can be fluid and may change as specific INPAC activities are implemented. Hospital champions also work with team members on their hospital units to make improvements to nutrition care based on INPAC and the malnutrition standard. The AMC program provides champions with resources and tools (eg, spreadsheet to collect audit data with automated feedback graphs) to support implementation of improved practices. Champions work with their site teams and are encouraged to use the Plan-Do-Check-Act cycle [35] to implement improved nutrition care practices, following a schedule that is feasible for their unit. At the start of the AMC program, champions typically concentrate their implementation efforts on nutrition risk screening and nutrition assessment for those screened at risk, as these practices drive all other nutrition care within INPAC [14,16]. When nutrition risk screening and assessments are routinely integrated into patient care, implementation efforts are focused on other INPAC activities (eg, food intake monitoring), while still conducting occasional checks to make sure screening is maintained.

#### Community of Practice

An online national community of practice occurs every 3-4 months and is hosted by the AMC leadership. The community of practice offers mentors and champions the chance to receive further education related to implementation and connect with peers across the country to foster continued motivation for the program. The community of practice is also an opportunity to address common challenges, promote the implementation of further INPAC activities, and discuss strategies for sustainable malnutrition care practices amongst a group of like-minded individuals. Topics discussed at community of practice sessions evolve based on challenges and needs identified by mentors and champions.

#### Anticipated Time Commitments and Timelines

There are multiple factors that can largely influence the time it takes to implement nutrition care practices in a hospital setting, including staffing restraints, organizational priorities and culture, barriers, facilitators, and baseline nutrition care practices. The fluidity of these factors is a barrier to precisely determining the time commitment affiliated with the mentor and champion roles. In addition, the role of mentors and champions is likely to evolve as the program ages. In general, implementation of nutrition risk screening and standardized nutrition assessment is expected to occur over a duration of 12-18 months.

The AMC program began recruiting mentors in April 2022 and champions in September 2022. The first community of practice was held in September 2022 and was structured based on previous implementation work from the More-2-Eat study [17]. The AMC program is anticipated to continue for several years with new hospitals, champions and mentors joining over the time. AMC leadership expects that timeline flexibility will be needed to accommodate shifting priorities, staffing restraints, barriers, and reduced hospital capacity secondary to public health emergencies or other constraints.

#### AMC Program Evaluation

A prospective, longitudinal, convergent parallel mixed methods design is being used to evaluate the AMC program [36]. The COM-B model is built into many of the data collection tools and strategies used in AMC (eg, interview guide designed to understand mentors’ and champions’ experiences) and will be used to evaluate behavior change, including change over time. Kirkpatrick’s 4-level framework will also be used to assess mentor and champion barriers to change by measuring their reaction to the training, learning of best practices, use of implementation strategies, and practice changes made [37]. Mentors and hospital champions are the primary participants in this program evaluation. Patient-level data that will be used for chart audits to assess the impact of improved nutrition care practices will be obtained by the hospital champion and transmitted to the AMC program team in an anonymized aggregated format, thus, patients are not considered “participants” in this program evaluation.

#### Baseline Data Collection

After AMC program training, mentors and hospital champions complete a baseline survey before the mentor-champion pairing and implementation of nutrition care practices (Table 1). Data collection is completed through a secure on-line platform (Qualtrics). The baseline surveys take approximately 30 minutes to complete and are voluntary. Mentors and hospital champions have the option to skip over any questions that they prefer not to answer. Hospital champions also complete a survey to describe their hospital and baseline nutrition care practices. This survey takes approximately 10 minutes.

After baseline surveys are complete, hospital champions complete a unit audit to determine nutrition care activities of patients since admission. Ideally, the chosen units will have an engaged health care team interested in implementing improved nutrition care practices and a minimum of 20 beds with longer stay patients (≥3 days) who are suitable for prevention, diagnosis, and treatment of malnutrition (ie, nonpalliative, pregnant, or postpartum). One weekday (eg, Tuesday) is selected as the audit day and data collection for the audit is conducted over a short time period (ideally 1-2 days) to capture current nutrition care practices. All patients on the chosen unit on the audit day are to be included in the audit process. Resources including an audit tool and audit tracking spreadsheet are provided by AMC leadership to facilitate the audit process. Alternatively, champions are encouraged to use their electronic medical record to populate an audit report, if available. Results from the audit are for internal hospital use only and are not shared with the AMC leadership. Rather, aggregated data from the unit audit will be transmitted to the AMC program in the form of an audit summary (eg, number screened at admission). It is anticipated that the unit audit will take up to 8 hours to complete if done by hand and submitting the audit summary through the online platform will take 10 minutes. Hospitals that have electronic medical records and can pull some or all of these data routinely will use this method for the audits. Hospital champions are encouraged to share these results with their implementation teams and site leadership to initially demonstrate a reason for change, and then to demonstrate if their change efforts are having an impact. Data collected aim to be beneficial for individual hospitals while the aggregate data deepen our understanding of the overall effectiveness of the AMC program.

**Table 1:** Overview of data collection tools used in evaluation of the Advancing Malnutrition Care program.

Data collection tool					Baseline						Quarterly						Annually			
					Mentors			Champions			Mentors			Champions			Mentors			Champions
**Experience survey**																				
	**Demographics**																			
		Age group, gender, racial background, professional role, length of time working in the hospital, and geographical region	✓			✓														
	**Implementation experience**																			
		Implementing nutrition care practices and using a mentor-champion model	✓			✓														
	**Behavior change**																			
		Measures of capability, opportunity, motivation, and confidence in being a mentor or champion	✓			✓			✓			✓								
**Hospital details survey**																				
	Hospital and unit(s) names, unit type(s), bed capacity, and staffing details						✓													
	**Implementation status of nutrition care best practices related to INPAC^a^**																			
		Status of screening, assessment, weight and food intake monitoring, use of energy-dense or protein-dense supplement with medication, and discharge planning				✓														
**Unit audit summary**																				
	Number of patients: in the audit, screened for nutritional risk, at nutritional risk, with a nutritional assessment (ie, SGA^b^) completed, who scored SGA A, B and C, with food intake monitoring recorded, and with body weight measured within 48 hours of admission						✓						✓							
	Average timing of: SGA from screening (in days), nutritional screening from time of hospital admission						✓						✓							
	Frequency of specific actions taken to improve nutrition, that is, nutritional interventions						✓						✓							
**Site progress survey**																				
	Summary of implementation activities undertaken on their units												✓							
Semistructured interviews																	✓			✓

^a^INPAC: integrated nutrition pathway for acute care.

^b^SGA: subjective global assessment.

#### Quarterly Data Collection

Approximately 3 months after baseline data collection, and quarterly thereafter, AMC leadership sends the mentor or champion a reminder to repeat the experience survey to solicit changes in their experiences. Hospital champions also complete a site progress survey and a summary of implementation activities undertaken to track site implementation progress (Table 1).

#### Qualitative Data Collection

The baseline hospital champion and mentor surveys provide an opportunity for the participant to indicate interest in being part of a future interview. One year after the start of implementation and approximately yearly afterward, select interested mentors and hospital champions may be asked to take part in an interview to gain a deeper understanding of their experiences with the AMC program and learn of strategies that they feel would enhance the program. Specifically, topics center on the mentor-champion relationship, COM-B, reimplementation, and program sustainability and scale (Multimedia Appendix 1). Due to the potential number of mentors and champions participating in AMC, all who express interest in participating in an interview may not be invited to do so. Interview participants will be based on trying to attain regional, hospital, and champion diversity. Mentors and champions will complete a separate informed consent process if invited to participate in an interview. Interviews will be conducted virtually or by phone and are expected to last 30-45 minutes. Closed captioning transcription will be used for web-based interviews and telephone audio files will be transcribed by a member of the research team or an independent professional transcriptionist.

### Statistical Analyses

Analyses described herein are related to the stated research questions; it is anticipated that additional secondary analyses will be completed. Briefly, analyses will include descriptive summaries (eg, means, SDs, and frequencies) of baseline mentor, champion, and hospital characteristics and mentor and champion experiences. Friedman test will be used to describe changes in mentors’ and champions’ experiences and their capability, motivation, and opportunity for implementing change over time (eg, at baseline and quarterly thereafter). Implementation of nutrition care practices will be summarized and compared between time points (eg, baseline and final audit) using chi-square tests to compare the proportion of patients who received selected nutrition care practices (eg, screening, weight monitoring, food intake monitoring) to provide insight into the sustainability of practices implemented. All data will be in aggregate form and no participant will be identifiable. Quantitative analyses will be conducted using SPSS (version 28.0.1.1; IBM Corp), GraphPad Prism Version 10.0.2, and Excel (Microsoft Corp).

Transcribed interview data will be checked by the interviewer [KF]. The COM-B model [32] will be considered when deductively coding data line-by-line. Directed content analysis [38] will guide the process of using the COM-B model to develop themes, which will be informed, described, and supported with quotes from the data. A summary of emerging themes will be reviewed with a minimum of 2 other researchers and will be mapped to the Behavior Change Wheel [32] to describe mentors and champions experiences with the AMC program and inform any changes needed. Qualitative data analyses will be conducted using Excel and NVivo 14. All data will be stored in a secure Faculty of Health server for use by the Nutrition and Aging Lab. VPN access is required to access this server. Folders and files will be encrypted and only available to AMC program evaluation investigators.

### Ethical Considerations

This study is approved by the University of Waterloo Human Research Ethics Board (ORE 44153). Written informed consent is obtained from mentors and hospital champions before data collection. Mentors and hospital champions create and submit a unique 9-digit identifier to be used during the data collection process to link responses while maintaining anonymity. Participating hospital sites determine on an individual basis if local research ethics board approval is required for hospital champions’ participation in the AMC program.

Prospective mentors and hospital champions who indicate interest in the AMC program will receive an electronic package that includes a detailed written and video description of the AMC program, a study information letter, and a consent form. Consenting champions will receive an electronic package that includes a memorandum of understanding and a data sharing agreement that is signed by the hospital site and the University of Waterloo. This process ensures that the hospital understands data collection that will occur, and how the hospital will be acknowledged in publications that result from the AMC program.

## Results

Data collection started in September 2022 and is projected to be completed by June 2025. As of August 2024, a total of 19 mentors and 46 champions had enrolled in the AMC program. Data analysis is projected to begin in July 2025, and results are expected to be published in 2026.

## Discussion

### Anticipated Outcomes

Evaluation of the AMC program is anticipated to inform the applicability of a mentor-champion model to spread and scale implementation of nutrition best care practices in Canadian hospitals. To date, few studies have observed, documented, and evaluated the process of spread and scale of a health innovation using a web-based mentor-champion program. To our knowledge, this is the first study to document and report on the national spread and scale of a malnutrition care initiative using a mentor-champion model. The documented success of 10 diverse hospitals implementing change to prevent, detect and treat malnutrition [15-17] suggests feasibility for spread and scale across Canadian hospitals.

The focus of the AMC program is on nutrition risk screening, assessment, and intervention in a hospital setting. The AMC program is unique as it is training mentors to support regional site hospital champions in their implementation efforts whereas mentorship in the More-2-Eat studies was provided through central administration [26]. The mentorship and implementation skills developed by all those involved in the AMC program also aim to boost confidence in how to be a mentor, how to change practice, and ultimately, how to make a difference in their own hospital and across the country. The AMC program aims to support hospital teams across Canada to address critical challenges related to hospital-based malnutrition, and ultimately improve nutrition care for patients across the country. Several of the educational goals (eg, improving clinical performance, recommending practice change, and offering decision-making support) align with the goals of academic detailing [39], and may provide an additional lens through which to consider the evaluation of this work. In contrast to the evaluation outlined herein, academic detailing conducts evaluation beyond changes in the decision-making process and also assesses the impact of education on patient-centered outcomes and resource use [39]. These are evaluation metrics that should be considered in future work and supplemented with data collection methods that capture the patient perspective (eg, interviews) on the impact of improved nutrition care on their stay.

### Strengths and Limitations

A strength of the AMC program and corresponding evaluation is the incorporation of models (eg, COM-B model [32]) and frameworks (eg, Kirkpatrick 4-level framework [37]) across stages of program development, implementation, and evaluation. Furthermore, the program includes hospital sites of various sizes, across Canadian provinces, and includes various in-patient units. These strategies support the generalizability of our findings, which will ultimately be determined based on the final number and demographics of participating sites. Our approach to program evaluation uses a mixed methods approach and will deepen our understanding of program effectiveness from both quantitative and qualitative perspectives. The lack of control sites for comparison during the evaluation process may be viewed as limitation although previous work in Canada has highlighted the prevalence of nutrition risk screening in hospital and has documented the need for improved implementation practices related to nutrition care [1,15-17,23].

### Conclusions

Evaluation of the AMC program will help support implementation of program changes aimed to positively impact nutrition care interventions. Mentors and champions are needed to implement sustainable nutrition care best practices, and this evaluation allows a deeper understanding of their experiences with the AMC program and will inform our ongoing efforts to spread and scale the AMC program, sustainably, across Canada. Ultimately, the lessons learned, and the tools developed by the AMC program and this evaluation, will aim to support optimized nutrition care for all patients.

## Appendix

Multimedia Appendix 1 Advancing Malnutrition Care mentor and champion interview guide.

## Abbreviations

AMC: advancing malnutrition care
COM-B: capabilities, opportunities, and motivations of behavior
INPAC: Integrated nutrition pathway for acute care

## Data Availability

The datasets generated during and/or analyzed during this study will be available from the corresponding author on reasonable request.
