# Supplementary material for: Spread and Scale of the Integrated Nutrition Pathway for Acute Care Across Canada: Protocol for the Advancing Malnutrition Care Program
Source: JMIR Res Protoc. 2024 Dec 31;13:e62764. doi: 10.2196/62764 (PMC11733522; doi:10.2196/62764)
Supplement: Multimedia Appendix 1 [file resprot_v13i1e62764_app1.pdf]

AMC Champions & Mentors – Interview Questions

Mentor/Champion Relationship

1. Can you tell me about your experience being a nutrition care [champion/mentor]?

   Probes:
   a. Why did you decide to become a nutrition care [champion/mentor]?
   b. How did it go at the beginning?
   c. Has your role changed over time?

2. Can you tell me about your experience with your [champion/mentor]?

   Probes:
   a. What topics do you typically cover in your conversations?
   b. What do you find useful about these conversations?
   c. Are [you/the champion] able to apply what you discuss? How?
   d. Can you provide an example of when [you/the champion] applied what you discussed in one of those conversations?
   e. How could these conversations be improved?
   f. *Mentor Only:* Do you ever apply what is discussed in these conversations to your own hospital?

3. What are the factors that you think contribute to a strong mentor-champion relationship? (trust etc.)

4. Have you attended any of the online Community of Practice events?
   a. If yes,
      i. Were you able to apply any of the learning from that event?

   b. How could these events be improved?

COM-B

5. In the online surveys, we ask you about your *capability* (i.e., skills, personality, etc.) to be a nutrition care [champion/mentor].
   a. What capabilities do you find are most helpful in this role? *Prompt:* organization; change management; optimism
   b. How have your capabilities changed during your time as a [champion/mentor]?
   c. Can you give me an example of how you have applied these capabilities/skills?
   d. Is there any anything else that AMC can do to support your capabilities? *Prompt:* more training?

6. In the online surveys, we ask if have the appropriate *opportunities* (i.e., time, organizational support) to be a nutrition care [champion/mentor].

a. What opportunities do you feel have supported you in this role? *Prompt:* hospital leadership support, CMTF support etc.
b. What system level factors have made your role easier? More difficult? (nutrition policy; new malnutrition standards etc.)?
c. How has your level of opportunity changed as a result of your participation in the AMC initiative?
d. Is there any anything else that AMC can do to support your opportunities?

7. In the online surveys, we ask about your *motivation* to be a [champion/mentor].
    a. What was your initial motivation for being a [champion/mentor]? How has this changed over time?
    b. Do you feel you have the *motivation* to continue as a nutrition care champion for the next few months? Next few years? Why/why not?
    c. Is there any anything else that AMC can do to support your motivation?

8. *Champions Only:* In the future, would you consider become a regional mentor?
    a. Why/why not?
    b. What would support your transition to this new role?

Re-Implementation

9. Many hospitals across Canada have been making improvements to nutrition care practices for the past several years, but not all of these changes continue long term. Many of these changes/initiatives stopped during the COVID-19 pandemic or for other reasons.

    a. Are there any nutrition-related initiatives (screening, volunteers etc.) that stopped in your hospital due to the pandemic or other reasons? If so:

        i. Why did they stop?
            1. Do you think they were implemented in a way that *could* have continued? Why/why not?
            2. Do you think the initiative was appropriate in the first place? (i.e. if not appropriate, there was a good reason to stop the initiative)
            3. Do you think the initiative was well implemented initially, but just not sustained? Why do you think it was not sustained?

        ii. Was the initiative restarted or are there efforts underway to restart?
            1. *If yes*, can you talk me through those re-starting efforts?
                a. How did it differ from the first time it was implemented?
                b. Did you follow any implementation plans?
                c. Who was involved? Was this different than the original implementation?

            If no, can you talk me through what you *would* do if these were to restart?

[Re-ask questions if more than one initiative was stopped.]

iii. Were any additional efforts made to make the changes more sustainable while restarting these initiatives? If yes, what efforts? If no, what else could be done to make them more sustainable?

AMC Program Sustainability & Scale-up

10. If it works, we want this mentor/champion program to continue long term. What do you think we need to do or consider in keeping this program going?

11. If it works, we also want to continue to expand across the country. What do you think we need to do to support this expansion?

12. By focusing on this champion approach, do you think there is anyone or any opportunities we are missing, or not supporting to make nutrition care improvements? What would be needed to support those individuals/hospitals/opportunities?

13. Is there anything else you think we should know about your role as a [champion/mentor]? About making nutrition care improvements in your hospital?
